# VN Quantum Dots Anchored onto Carbon Nanofibers as a Superior Anode for Sodium Ion Storage

**DOI:** 10.3390/ma17236004

**Published:** 2024-12-07

**Authors:** Xiaoyu Wu, Haimin Zhang, Jiachen Yanghe, Sainan Liu

**Affiliations:** 1 School of Minerals Processing and Bioengineering, Central South University, Changsha 410083, China; 2 Hunan Zoomlion Neo Material Technology Co., Ltd., Changsha 410083, China

**Keywords:** VN, quantum dots, anode, sodium-ion batteries

## Abstract

Vanadium-based compounds exhibit a high theoretical capacity to be used as anode materials in sodium-ion batteries, but the volume change in the active ions during the process of release leads to structural instability during the cycle. The structure of carbon nanofibers is stable, while it is difficult to deform. At the same time, the huge specific surface area energy of quantum dot materials can speed up the electrochemical reaction rate. Here, we coupled quantum-grade VN nanodots with carbon nanofibers. The strong coupling of VN quantum dots and carbon nanofibers makes the material have a network structure of interwoven nanofibers. Secondly, the carbon skeleton provides a three-dimensional channel for the rapid migration of sodium ions, and the material has low charge transfer resistance, which promotes the diffusion, intercalation and release of sodium ions, and significantly improves the electrochemical activity of sodium storage. When the material is used as the anode material in sodium ion batteries, the specific capacity is stable at 230.3 mAh g^−1^ after 500 cycles at 0.5 A g^−1^, and the specific capacity is still maintained at 154.7 mAh g^−1^ after 1000 cycles at 2 A g^−1^.

## 1. Introduction

Lithium-ion batteries, as the main electrochemical energy storage devices in the market, have limited future prospects due to the scarcity of lithium resources [1]. Therefore, researchers have started evaluate similar elements such as sodium and potassium, on account of their abundant reserves [2,3,4]. Recently, sodium-ion batteries have started to undergo commercial mass production. However, there is a serious problem that has not been well addressed. This is the change in the volume of the material during intercalation and release due to the larger radius of sodium ions [5]. It is exceedingly challenging to regulate the topology and crystalline structure of the electrode materials, thereby complicating the design of battery systems [6,7].

In order to address this issue, active anode materials such as carbon [8,9] metals and their alloys (Sn [10], Sb [11], Ge [12], SnSb [13,14]), metal oxides (SnO_2_ [15,16], TiO_2_ [17,18]) and transition metal dichalcogenides (MoS_2_ [19], NbS_2_ [20], VS_2_ [21]) with structural stability have been particularly effective. Among them, as a mainstream commercial battery anode material, the specific capacity of carbon limits its further development. Common methods for improving the performance of anode materials include atom doping [22], coating protection [23], structural nanoization [24], and the construction of composite materials [25]. The utilization of anode materials at the quantum dot level not only serves to enhance ionic conductivity, but also facilitates the attainment of a substantial specific surface area, thereby accelerating the reaction rate. Quantum dots (QDs), as an emerging zero-dimensional material, exhibit unique quantum effects such as the quantum tunneling effect, the quantum limiting effect, and the surface effect [26,27,28] due to their unique structure (size < 10 nm), and are widely used in fields such as catalysis [29], optical sensing [30], biomedicine [31], and solar cells [32]. VN is a safe and stable vanadium-based compound with high metal conductivity and high theoretical capacity. In the process of sodium storage, in addition to the formation of SEI films, Na_3_N and V will inevitably be generated, and the resulting volume expansion will greatly hinder the ideal electrochemical performance of VN, which makes its performance poor. Not only is the structure of the carbon nanofiber stable, but the carbon skeleton also provides a three-dimensional channel for the rapid migration of sodium ions. To sum up, we couple VN quantum dots and carbon nanofiber together so that they play to their strengths respectively. Therefore, we make VN quantum dots couple on carbon nanofiber via electrostatic spinning and heat treatment, which make it structurally stable and have excellent electrochemical properties.

Herein, we fabricate carbon nanofiber–VN quantum dots (VN/CNF) that have a fibrous network structure, in which VN quantum dots with sizes of smaller than 10 nm are homogeneously dispersed. The distinctive structural and morphological characteristics enable the effective mitigation of volume alterations in VN, preventing their aggregation throughout the cycle processes. Additionally, they enhance electrical conductivity and facilitate the diffusion, intercalation and release of sodium ions. Finally, VN/CNF, as an anode material for SIB, shows high-rate capabilities, excellent long-term cycle performance and high capacities.

## 2. Materials and Experimental Characterization Methods

### 2.1. Synthesis Details

First, 1 g of acetylacetonate vanadium (Shanghai Aladdin Biochemical Technology Co., Ltd., CP, Shanghai, China) was mixed with 6 mL of DMF (Sinopharm Chemical Reagent Co., Ltd., AR, Shanghai, China), then magnetically stirred using the LED digital magnetic stirrer (MS-PA, Beijing Dalong Xingchuang Experimental Instrument Co., Ltd., Beijing, China) for 30 min. Then, 0.4 g of polyacrylonitrile (PAN) (Sigma-Aldrich, Burlington, MA, USA), with a molecular weight equal to approximately 150,000, was dissolved in a 10 mL beaker containing 4 mL of N, n-dimethylformamide (DMF) and magnetically stirred for 30 min. The two solutions were combined and subjected to sonication (JP-040, Shenzhen Jie Meng cleaning equipment Co., LTD., Shenzheng, China) until a homogeneous dispersion was achieved. A solution of 10 mL was then absorbed by a syringe for the purpose of electrospinning (YFSP-T, Tianjin Yunfan Technology Co., LTD., Tianjin, China). The aluminum foil was covered on the receiver to collect the subsequent electrospinning products, and the distance between the aluminum foil and the tip of the syringe was controlled at 15 cm, while the syringe was pushed at 0.002 mm s^−1^. Then, the nitride precursor was prepared by electrospinning in the high-temperature atmosphere tube furnace (OTF-1200X-S, Hefei Kejing Material Technology Co., Ltd., Hefei, China) at 550 °C for 2 h in an ammonia atmosphere (heating rate, 5 °C min^−1^). VN/CNF was produced by allowing the material to cool to room temperature and then grinding it completely. The preparation of vanadium nitride (VN) invovled the addition of nitride to an appropriate amount of vanadium oxy acetylacetonate under the same heat treatment conditions, with VN obtained after grinding.

### 2.2. Characterization Techniques

The qualitative evaluation of the prepared material was conducted using an X-ray diffractometer (TTR III) with Cu Kα radiation (λ = 1.54184 Å) in the 5°∼80° range. The chemical composition of the sample was tested by an X-ray photoelectron spectrometer (XPS). In an air atmosphere (TGA, NETZSCH STA449F3, Shanghai, China), the material was subjected to thermogravimetric analysis at a heating rate of 10 °C min^−1^ to obtain the carbon content of the material. Scanning (Nova Nano SEM 230, FEI Company, Hillsboro, OR, USA) and transmission electron (FEI Tecnai G2 F20, FEI Company, Hillsboro, OR, USA) microscopy (SEM and TEM, respectively) were used to analyze the morphologies and structures of the materials, respectively.

### 2.3. Electrochemical Measurements

VN/CNF and VN were mixed with Super P and PVDF at a ratio of 8:1:1, and an appropriate amount of NMP was added. The serum bottle was kept in a sealed state and magnetically stirred for 24 h to obtain an evenly dispersed slurry. After mixing, the two types of slurry were coated on the copper foil with a plate coating machine (MSK-AFA-SC200, Shenzhen Kejing Zhida Technology Co., Ltd., Shenzhen, China) and dried in the oven (DHG-9076A, Shanghai Jinghong Experimental Equipment Co., Ltd., Shanghai, China) overnight. The SIB anodes, which were 12 mm in diameter, were prepared by punching with a punch–die (MSK-T10, Hefei Kejing Material Technology Co., Ltd., Hefei, China). Half of the batteries were assembled for subsequent tests using CR2016 coin cell casings in a glovebox (MB-BL-08, MBRAUN GmbH Co., Ltd., Garching, Germany) with a water oxygen content of less than 0.1 ppm; this was in the order of sodium sheet, electrolyte, diaphragm, electrolyte, and electrode sheet. The glass fiber membrane was used as a diaphragm, and 1 M of NaPF6 was dissolved in a mixture of vinyl carbonate (EC)/dimethyl carbonate (DMC) with a volume ratio of 1:1, and 5 vol% fluorinated vinyl carbonate (FEC) was added as the SIB electrolyte. Electrochemical tests were performed on the CT2001A battery tester (LAND Electronic Co., Wuhan, China) and electrochemical workstation. In particular, a cyclic voltammetry test was conducted at a sweep speed of 0.1 mV s^−1^ (0.01 to 3 V vs. Na^+^/Na). The EIS was conducted within the frequency range of 0.01 kHz to 100 kHz. Both experiments were conducted on a CHI-600E workstation (CHI-600E, Shanghai, China).

## 3. Results and Discussion

### 3.1. Analysis of the Structure of VN/CNF and VN

**Figure 1:**
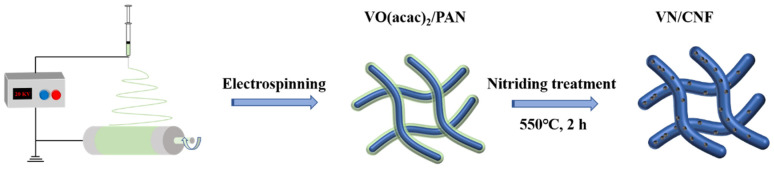
Schematic synthesis steps of VN/CNF.

Figure 1 shows the synthesis process of VN/CNF in detail. The precursor material with a unique 3D support structure was obtained by the electrospinning method, and vanadium acetyl acetonate containing vanadium oxide was introduced into the spinning process and further nitrided in the subsequent heat treatment step; finally, a unique VN/CNF was obtained.

**Figure 2:**
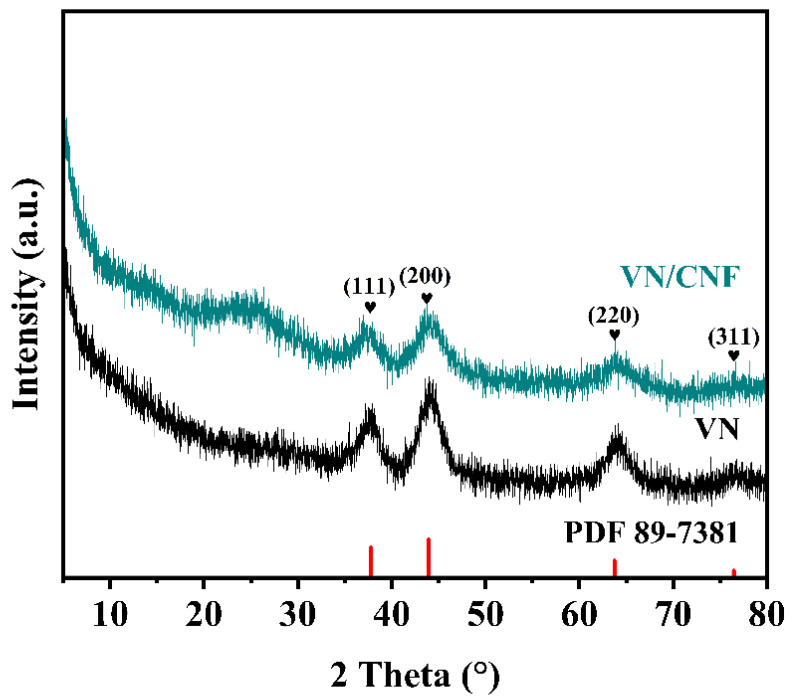
XRD patterns of VN/CNF and VN.

The composition and structures of the VN/CNF and VN samples were characterized by X-ray diffraction (XRD). As illustrated in Figure 2, the diffraction peaks of VN/CNF and VN at 27.6°, 43.7°, 63.6°, and 76.3° can be effectively aligned with the standard PDF card 89-7381, which corresponds to the (111), (200), (220), and (311) crystal planes of VN, respectively. In comparing VN with VN/CNF, the former displays a protruding drum peak at approximately 25°; this peak aligns with the carbon fiber skeleton obtained through the heat treatment process of PAN.

**Figure 3:**
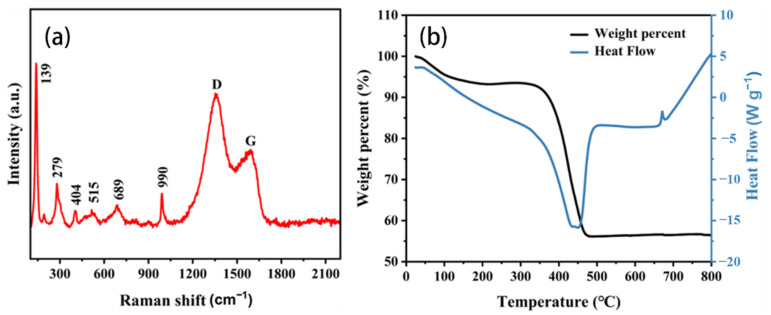
(**a**) Raman spectra and (**b**) TG-DSC curves of VN/CNF.

The Raman spectrum in Figure 3a further proves the authenticity of this conclusion. Figure 3a shows the Raman spectrum of VN/CNF. Figure 3a illustrates the presence of two distinct peaks at 1364.0 cm^−1^ and 1590.9 cm^−1^, which can be identified as the D peak and the G peak of the carbon material, respectively. This evidence substantiates the presence of carbon in the composite material under investigation. According to the calculation, I_D_/I_G_ = 3619.7/2067.1 = 1.75, which indicates that there are many defects in this carbon material [33].

From Figure 3a, it can be seen that 139 cm^−1^, 279 cm^−1^, 405 cm^−1^, 515 cm^−1^, 689 cm^−1^ and 990 cm^−1^ correspond to VN. This is also shown in Figure S1. The results demonstrated the successful synthesis of VN/CNF and VN.

The TG-DSC test was performed within the range of 30~800 °C for VN/CNF and VN in order to analyze the content of the carbon materials (Figure 3b and Figure S2). Figure S2 illustrates that the mass loss observed prior to 240 °C was primarily attributable to the volatilization and adsorption of water in the VN and that VN was converted into V_2_O_5_ in the subsequent reactions. Accordingly, in Figure 3b, in addition to the volatilization of the adsorbed water, the carbon oxidation in the VN/CNF is CO_2_ and the rest of VN from VN/CNF is converted to V_2_O_5_. The calculation results show that the content of carbon in VN/CNF is about 59.66%.

**Figure 4:**
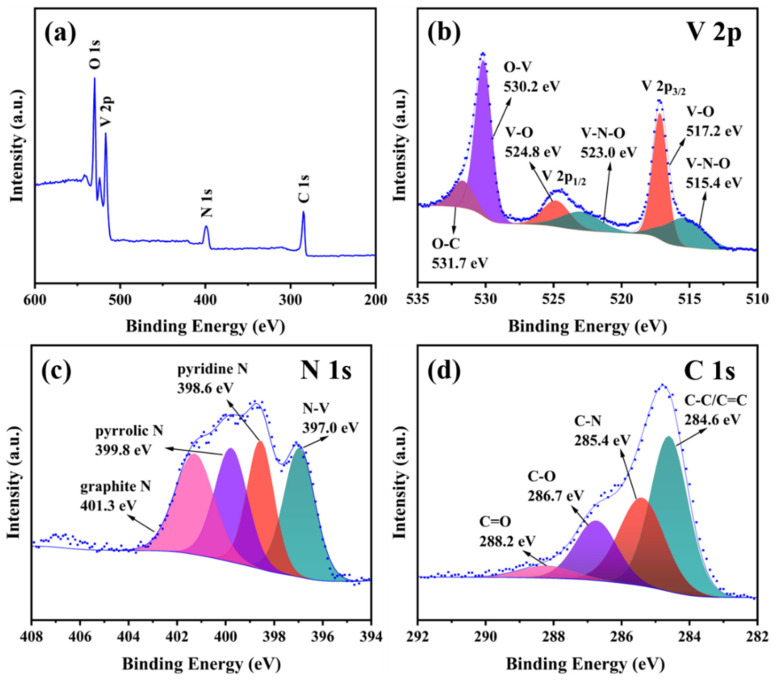
(**a**) Survey and high-resolution (**b**) V 2p, (**c**) N 1s and (**d**) C 1s spectra recorded for the VN/CNF.

Figure 4 is the XPS diagram of VN/CNF. Figure 4a illustrates the presence of C, N, O, and V elements in the prepared VN/CNF. Figure 4b illustrates the presence of two distinct sub-peaks in the V 2p_3/2_ spectrum. These sub-peaks, observed at 515.4 and 517.2 eV, are attributed to V-N-O and V-O bonds, respectively [34]. Correspondingly, there are two peaks of V 2p_1/2_ peaks at 523.0 and 524.8 eV. The two peaks at 530.2 and 531.7 eV are related to the O-V bond and O-C bond. This shows that some oxidation occurs in nitride. The peak values of 397.0, 398.6, 399.8, and 401.3 eV correspond to N 1s, pyridine nitrogen, pyrine nitrogen, and graphite nitrogen (Figure 4c) of N 1s. The peaks at 284.6, 285.4, 286.7, and 288.2 eV, as shown in Figure 4d, are attributed to the C-C/C=C, C-N, C-O, and C=O bonds of the C 1s spectrum. XPS proves the generation of VN, which is combined with carbon fiber through the price bond.

**Figure 5:**
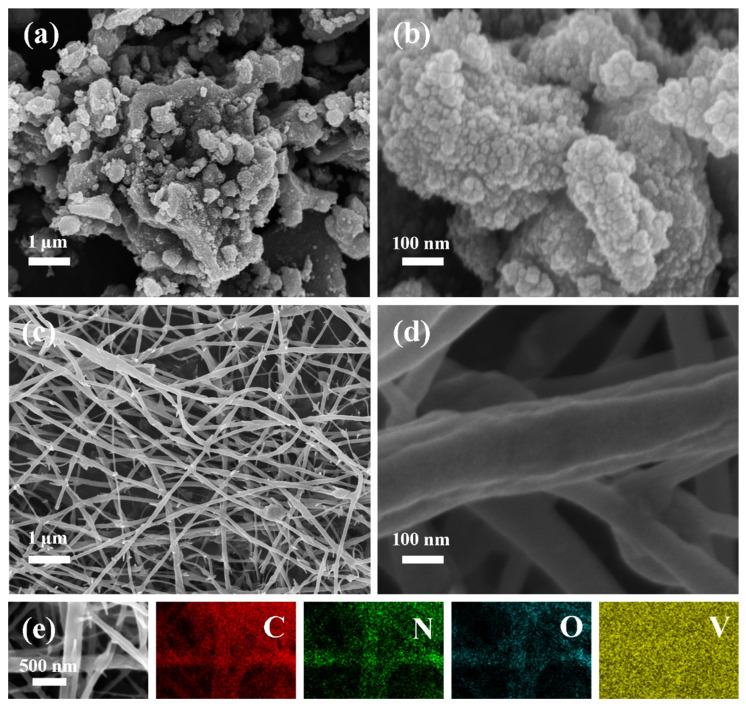
SEM image of (**a**,**b**) VN and (**c**,**d**) VN/CNF, (**e**) HAADF image, and corresponding elemental mapping distribution of VN/CNF.

**Figure 6:**
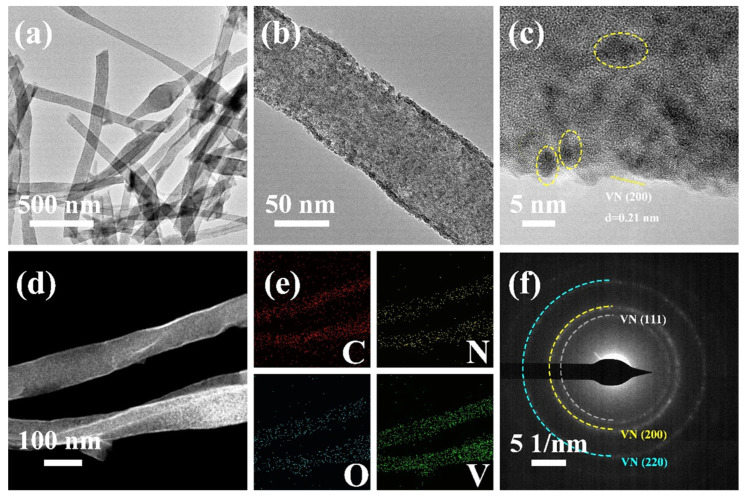
(**a**,**b**) TEM and (**c**) HRTEM image of VN/CNF and (**d**) HAADF image of VN/CNF. (**e**) the corresponding elemental mapping of C, N, O and V elements and (**f**) SAED images of VN/CNF.

The morphology and microstructure of the VN/CNF composite and the pure VN material were identified through the use of scanning electron microscopy (SEM) and transmission electron microscopy (TEM) (Figure 5 and Figure 6). From the SEM images of VN at different magnifications, it is obvious that VN agglomeration is serious and that the particle size is different. VN/CNF has a fibrous interlaced mesh structure, with the coupling of VN quantum dots (Figure 5c). Compared with VN powders with severe agglomeration, VN/CNF with an interwoven three-dimensional network has a higher specific surface area. This structure is conducive to the wetting of the electrolyte, which is expected to improve the electrochemical performance of the electrode material. After further magnification, it can be seen that the surface of a single fiber is not smooth, which may be due to the morphological changes caused by the nitriding of the V element in the raw material, which is attached to the surface of the carbon fiber (Figure 5d). Figure 5e shows the HAADF image of the composite material and the corresponding element distribution diagram. The distribution of the C, N, O and V elements is observable, occurring in a uniform manner on the surface of the carbon fiber. This result is also consistent with previous detection results.

Figure 6 shows the transmission image of VN/CNF. As can be seen from the figure, the surface of the fibrous sample presents an uneven state. The high-resolution transmission map further shows that many VN quantum dots are uniformly clustered on the surface of the carbon fiber (Figure 6b). In Figure 6c, the VN particles are marked with the yellow circles. Meanwhile, the 0.21 nm lattice spacing of the carbon fiber edge corresponds to the (200) crystal face of VN (Figure 6c). Furthermore, the HAADF diagram of VN/CNF and the corresponding mapping diagram of the C, N, O and V elements can be employed to illustrate the uniform distribution of the aforementioned elements in the VN/CNF (Figure 6d,e). The diffraction ring of the SAED image in Figure 6f correlates with the (111), (200), and (220) planes of VN.

### 3.2. Sodium Storage Performance Research

**Figure 7:**
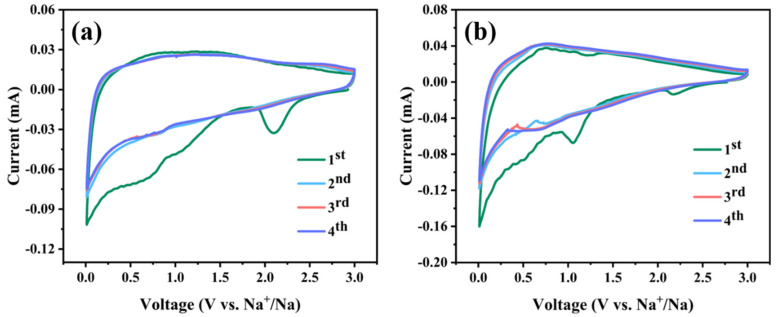
CV of (**a**) VN/CNF and (**b**) VN-containing anodes recorded at 0.1 mV s^−1^ and 0.2 mV s^−1^, respectively.

The electrochemical properties of VN/CNF and VN as SIB-negative materials are assessed by buckle batteries. Figure 7 illustrates the cyclic voltammetry curve of the VN/CNF and VN electrodes at a scanning speed of 0.1 mV s^−1^ and 0.2 mV s^−1^, respectively. In the initial cycle, three cathodic peaks are observed at 2.10, 1.06 and 0.7 V, which are not present in subsequent cycles. These peaks correspond to the formation of the solid electrolyte interface (SEI) film. The highly coincident CV curves from the second cycle indicate that the VN/CNF electrode exhibits remarkable electrochemical reversibility. In Figure 7b, there are also three sharp cathodic peaks at 2.17, 1.06 and 0.5 V, which indicates the formation of an SEI film. The coincidence of the subsequent cycle is worse than that for VN/CNF. The impedance data of VN/CNF and VN in the initial state were, respectively, tested (Figure S3). In the equivalent circuit shown in Figure S3, the symbols R_1_, R_2_, CPE and W are used to represent the solution impedance, charge transfer impedance, non-ideal double-layer capacitance and diffusion impedance, respectively. As illustrated in Figure S3, the charge transfer impedances of the VN/CNF and VN electrodes are 2265 and 3496 Ω, respectively, indicating more excellent kinetics. The enhancement of electrical conductivity in VN/CNF is facilitated by the incorporation of carbon nanofibers, which also serve to mitigate the effects of volume expansion and optimize the electrochemical performance of the material [35].

**Figure 8:**
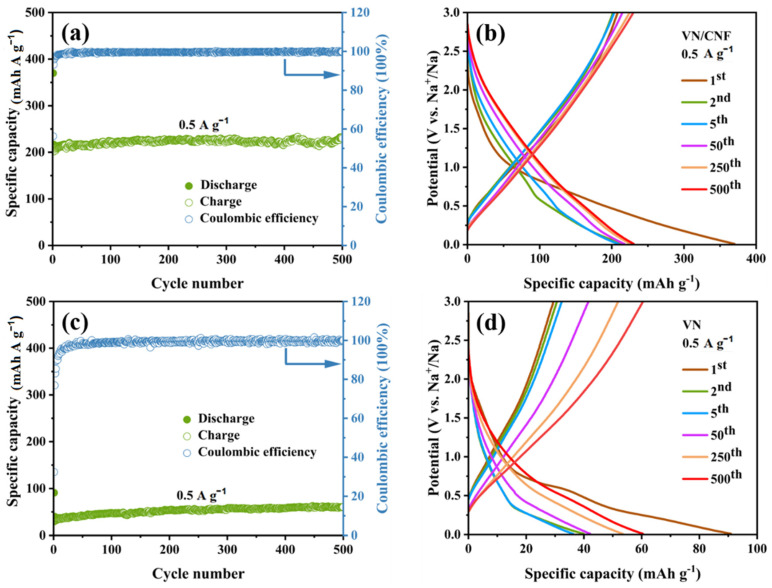
Cycling performance of (**a**) VN/CNF and (**c**) VN-based SIB at 0.5 A g^−1^; GCD curves of (**b**) VN/CNF and (**d**) VN anodes at 0.5 A g^−1^.

The performance of the battery with VN/CNF is significantly better than that with VN when cycling at a 0.5 A g^−1^ current density. As can be seen from Figure 8a, under this condition, the specific capacity of the SIB assembled based on VN/CNF is still 230.3 mAh g^−1^ after 500 cycles, while the VN electrode was only 60.4 mAh g^−1^ after 500 cycles (Figure 8c). A high reversible capacity of 369.9 mAh g^−1^ is achieved at a current rate of 0.5 A g^−1^ with VN/CNF (Figure 8b) in the first cycle. The value in consideration is markedly higher than that of VN (90.9 mAh g^−1^) (Figure 8d); its coulomb efficiency (56.4%) is also considerably higher than that of VN (32.4%). The lower coulomb efficiency in the first cycle of both materials implies a large capacity loss, mainly due to the formation of SEI films, which is consistent with the results of VN/CNF and VN in CV [36]. It is the structural advantage of VN/CNF that makes the coulomb efficiency higher, reducing the irreversible capacity loss of the battery.

**Figure 9:**
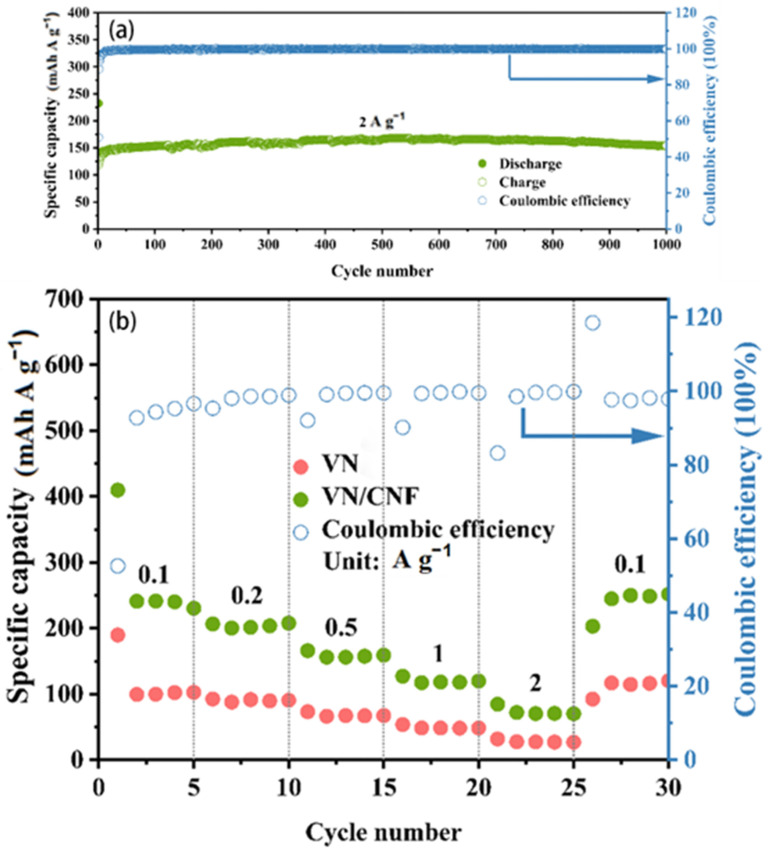
(**a**) Long-term cycling performance of VN/CNF-based SIB at 2 A g^−1^. (**b**) Rate capability of VN/CNF and VN-based SIB.

As illustrated in Figure 9a, the long-cycle performance of the battery with VN/CNF at a high current density is also excellent. The battery using VN/CNF can still maintain a specific capacity of 154.7 mAh g^−1^ after 1000 cycles at a current density of 2 A g^−1^. In addition, the battery with VN/CNF exhibits good rate capabilities at different current rates from 0.1 A g^−1^ to 2 A g^−1^ for sodium storage. After five cycles at different current densities, average specific capacities of 272.5, 203.9, 159.0, 120.2 and 73.6 mAh g^−1^ are reached, respectively. When the current density returns to 0.1 A g^−1^, the specific capacity is restored to 203.3 mAh g^−1^ (Figure 9b). The rate capability of the battery with VN is obviously inferior to that of VN/CNF. Compared with the VN/CNF anode, the average discharge capacity is only 118.9, 90.5, 68.3, 49.4, and 28.2 mAh g^−1^. When the current density returns to 0.1 A g^−1^, the specific capacity is only 92.5 mAh g^−1^. The enhanced rate performance can be attributed to the superior kinetics of VN/CNF, which exhibits a lower charge transfer impedance.

It is evident that a structural advantage is exhibited in the non-original SEM image of the VN/CNF anode and the VN anode at a current density of 0.5 A g^−1^. The morphology of the VN/CNF anode did not change significantly during the cycle, and there were many VN quantum dots coupled to the interwoven carbon nanofibers (Figure S4a,b), indicating the superiority of the material structure. Meanwhile, the large specific surface area of VN quantum dots allows them to fully contact the electrolyte and not agglomerate. On the contrary, the pure VN is clustered together and cannot fully contact the electrolyte (Figure S4c,d), so the high theoretical specific capacity brought about by the structural advantages of the VN itself cannot be used.

As a novel anode for vanadium-based sodium ion batteries, VN/CNF has a performance that is no lower than other anode materials used in vanadium-based sodium ion batteries, and exhibits great application prospects. The specific electrochemical performance is shown in Table 1.

## 4. Conclusions

A VN-quantum-dot-modified carbon nanofiber was reported, and this combines the excellent electrical conductivity of VN quantum dots, their wide and stable potential window, the structural stability of the carbon nanofiber, and the advantages of the mesh structure formed by interwoven nanofibers. It provides abundant channels for electron transfer in the negative electrode. The results show that compared with VN, VN/CNF is better when used as the anode in sodium ion batteries. A high capacity (500 cycles/230.3 mAh g^−1^) and excellent long-cycle stability (2 A g^−1^/1000 cycles/154.7 mAh g^−1^) can be obtained under the condition of 0.5 A g^−1^. The results demonstrated in this work show the feasibility and efficiency of modifying anode materials using quantum dot materials.

## Figures and Tables

**Table 1:** Electrochemical performance comparison of the VN/CNF with other V-based anode materials for SIB [4,37,38,39,40,41,42,43,44,45].

Sample	Current Density (A g^−1^)	Cycle Number	Capacity Retention (m Ah g^−1^)
SbVO_4_/G	0.1	450	401.6
B-SbSn/NCFs	0.1	400	486.9
VN/CNF	0.1	100	403
PbSe@CNTs	0.1	100	458.9
FVO/rGO	1	1500	137
VSe_1.5_/CNFs	2	2000	265
WVO_4_/V_3_Se_4_/CNFs	5	25,000	137
D-V_5_S_8_/CNFs	5	17,000	190
V_3_Se_4_/NPCNFs	10	13,000	240
SnSx-N/P-CNFs	10	32,000	214
**VN/CNF**	0.5	500	230.3
	2	1000	154.7

## Data Availability

The original contributions presented in the study are included in the article/Supplementary Materials, further inquiries can be directed to the corresponding authors.

## Supplementary Materials

The following supporting information can be downloaded at: https://www.mdpi.com/article/10.3390/ma17236004/s1, Figure S1. Raman spectra of VN. Figure S2. TG-DSC curves of VN. Figure S3. Nyquist plots of VN/CNF and VN as the anode materials for SIB before cycling. Figure S4. The ex situ SEM images of (a,b) VN/CNF and (c,d) VN-based SIB anodes after the 10th cycle at 0.5 A g^−1^.
