# Supplementary material for: VN Quantum Dots Anchored onto Carbon Nanofibers as a Superior Anode for Sodium Ion Storage
Source: Materials (Basel). 2024 Dec 7;17(23):6004. doi: 10.3390/ma17236004 (PMC11643878; doi:10.3390/ma17236004)
Supplement: Supplementary file 1 [file materials-17-06004-s001.zip › materials-3336788-supplementary.pdf]

materials

Supporting Information

VN Quantum Dots Anchored onto Carbon Nanofibers as a Superior Anode for Sodium Ion Storage

Xiaoyu Wu [1,†], Haimin Zhang [2,†], Jiachen Yanghe [1] and Sainan Liu [1,*]

[1] School of Minerals Processing and Bioengineering, Central South University, Changsha 410083, China; 235611138@csu.edu.cn
[2] Hunan Zoomlion Neo Material Technology Co., Ltd., Changsha 410083, China; zhanghaimin@csu.edu.cn (H.Z.); 8204211522@csu.edu.cn (J.Y.)
* Correspondence: lsn@csu.edu.cn
† These authors contributed equally to this work.

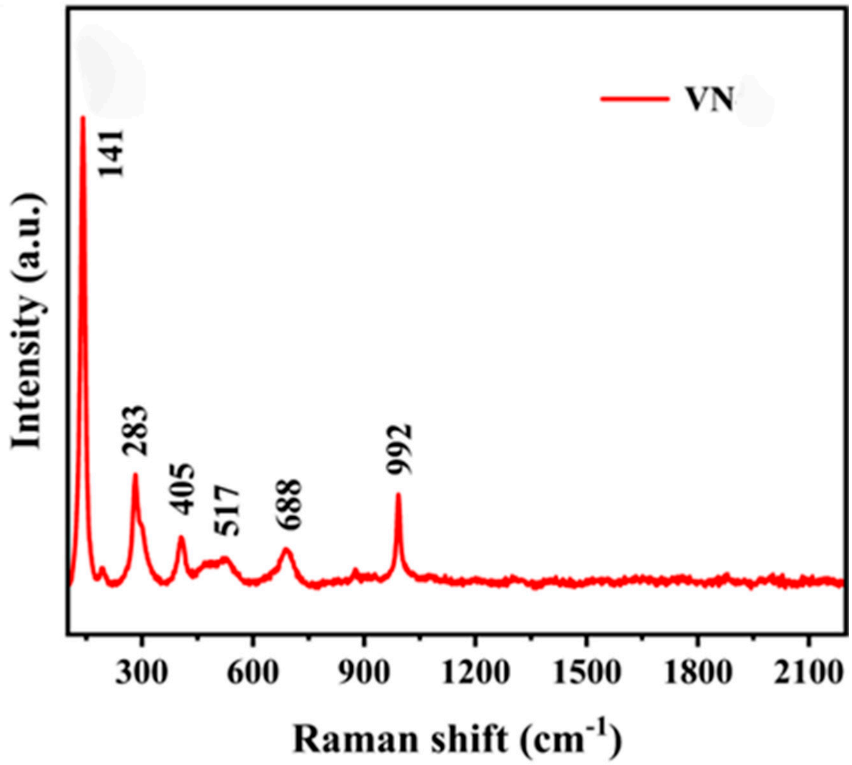

Figure S1. Raman spectra of VN.

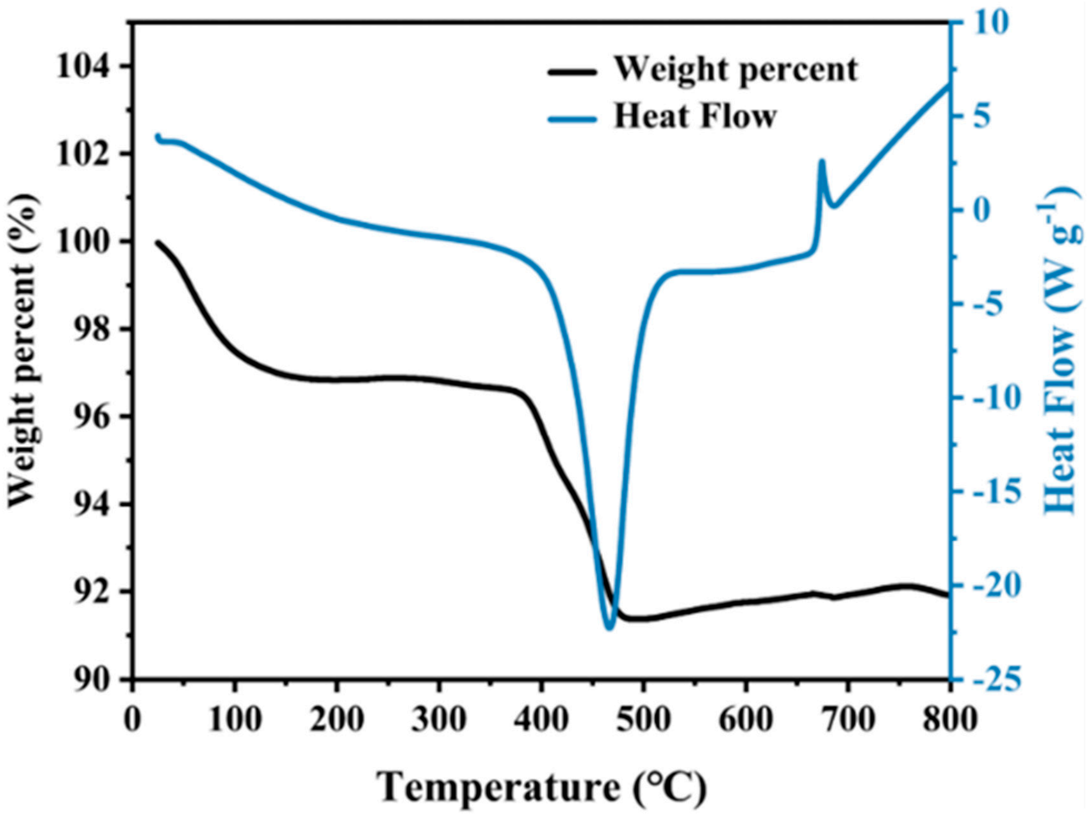

Figure S2. TG-DSC curves of VN.

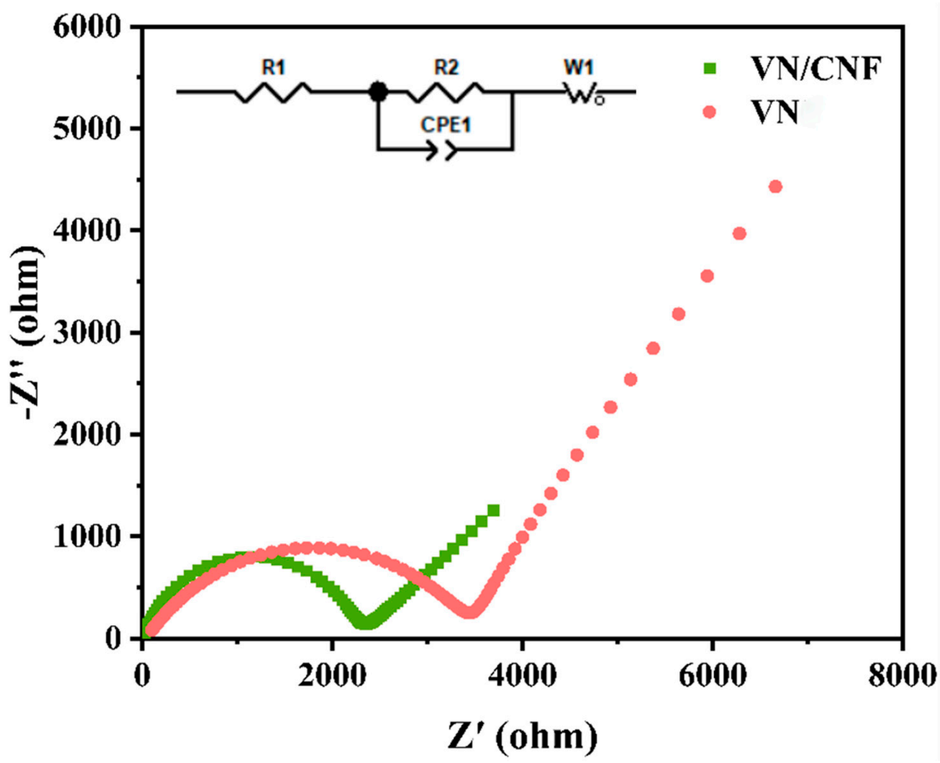

Figure S3. Nyquist plots of VN/CNF and VN as the anode materials for SIB before cycling.

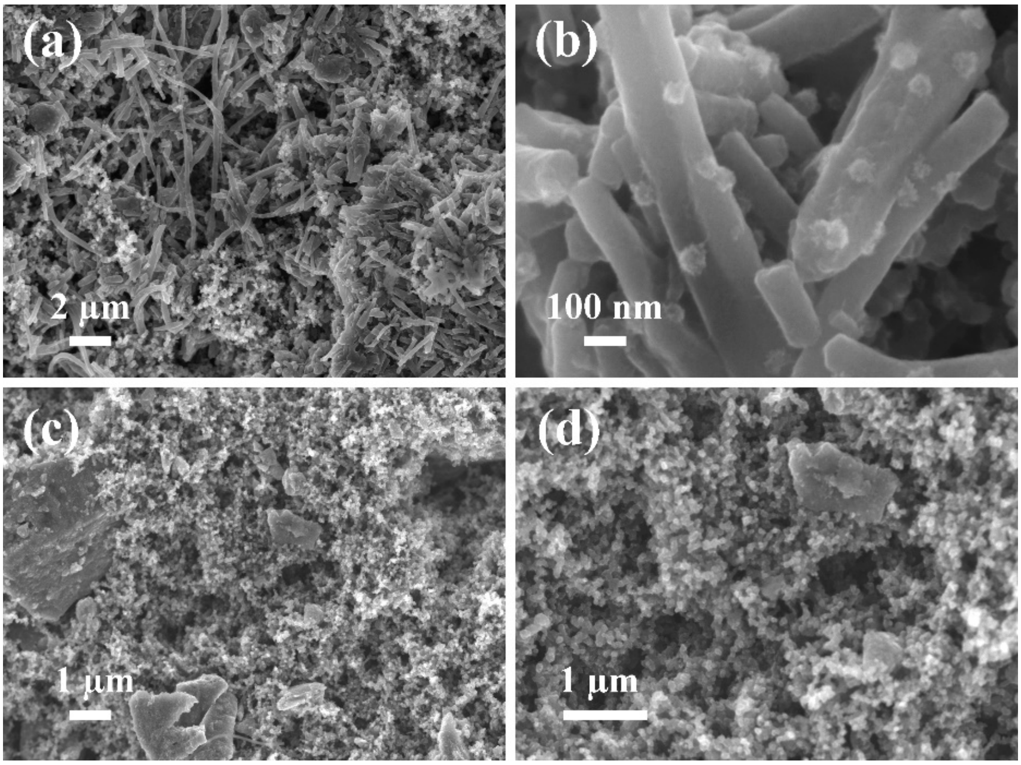

Figure S4. The ex situ SEM images of (a,b) VN/CNF and (c,d) VN-based SIB anodes after the 10th cycle at 0.5 A g^{-1}.
